# Kansas

**Published:** 1896-06

**Authors:** 


*Kansas.* The suit of the cattlemen against the Missouri, Kansas, and Texas Railroad Company, and shippers over the same, for shipping cattle into the grazing country and spreading Texas fever among their stock, has been decided at Topeka, and the cattlemen awarded $50,000 damages. This decision is by the Supreme Court, and sustains that of the lower court.

National Acting-Secretary Dabney, of the Department of Agriculture, has issued orders to all inspectors to see that all animals coming under their direction and control be properly fed, watered, and humanely treated by all employés and by all railroad, stock-yard, and other employés, to lessen the suffering losses, etc., incidental to the cattle-industry.

